# Enteropathogenic *Escherichia coli* (EPEC) expressing a non-functional bundle-forming pili (BFP) also leads to increased growth failure and intestinal inflammation in C57BL/6 mice

**DOI:** 10.1007/s42770-022-00802-5

**Published:** 2022-07-26

**Authors:** Solanka Ellen Ledwaba, David Thomas Bolick, Pedro Henrique Quintela Soares de Medeiros, Glynis Luanne Kolling, Afsatou Ndama Traore, Natasha Potgieter, James Paul Nataro, Richard Littleton Guerrant

**Affiliations:** 1 Department of Biochemistry and Microbiology, Faculty of Science, Engineering and Agriculture, University of Venda, Thohoyandou, Limpopo Province South Africa; 2 Division of Infectious Disease and International Health, School of Medicine, University of Virginia, Charlottesville, VA USA; 3 Department of Microbiology, Faculty of Medicine, Federal University of Ceara´, Fortaleza, Brazil; 4 Department of Biomedical Engineering, University of Virgina, Charlottesville, VA USA; 5 Department of Pediatrics, School of Medicine, University of Virginia, Charlottesville, VA USA

**Keywords:** Enteropathogenic *E. coli*, Bundle-forming pili, Murine model, Diarrhea, Inflammation

## Abstract

Bundle-forming pili (BFP) are implicated in the virulence of typical enteropathogenic *E. coli* (EPEC), resulting in enhanced colonization and mild to severe disease outcomes; hence, non-functional BFP may have a major influence on disease outcomes in vivo. Weaned antibiotic pre-treated C57BL/6 mice were orally infected with EPEC strain UMD901 (E2348/69 *bfpA* C129S); mice were monitored daily for body weight; stool specimens were collected daily; and intestinal tissues were collected at the termination of the experiment on day 3 post-infection. Real-time PCR was used to quantify fecal shedding and tissue burden. Intestinal inflammatory biomarkers lipocalin-2 (LCN-2) and myeloperoxidase (MPO) were also assessed. Infection caused substantial body weight loss, bloody diarrhea, and intestinal colonization with fecal and intestinal tissue inflammatory biomarkers that were comparable to those previously published with the wild-type typical EPEC strain. Here we further report on the evaluation of an EPEC infection model, showing how disruption of *bfp* function does not impair, and may even worsen diarrhea, colonization, and intestinal disruption and inflammation. More research is needed to understand the role of *bfp* in pathogenicity of EPEC infections in vivo.

## Introduction

Diarrhea continues to be a problem in young children especially those infected with enteropathogenic *E. coli* (EPEC) [1, 2]. The classification of enterohemorrhagic *E. coli* (EHEC) and EPEC (typical and atypical strains) pathotypes has resulted in a significant increase in knowledge of the epidemiology, pathophysiology, and clinical presentation of these pathogen infections [3–5]. Aside from their common virulence determinants, these pathotypes differ in their virulence depending on the presence or absence of bundle-forming pili (BFP), the EPEC adherence factor (EAF) plasmid, and Shiga toxin production [5–7]. EPEC causes moderate to severe diarrhea in children under 12 months of age [2]. Typical EPEC strains demonstrate distinct localized adherence in cell lines and in tissue biopsies [8, 9]. This distinct pattern is associated with protruding flagella and BFP which are thought to assist in contact to the host cells, followed by intimate adherence resulting in attaching and effacing lesions with actin accumulation at the site of infection [10, 11].

BFP is a type IV fimbriae [12] encoded by the EAF plasmid [13, 14]. It consists of a cluster of 14 genes, with the *bfpA* gene encoding the major adhesin subunit (bundlin) and the 13 other genes (*bfpB* to *bfpL*) involved in BFP biogenesis [15–18]. BFP is produced during bacteria-bacteria interaction resulting in a mesh-work of fibers that lead to microcolony formation, enhancing the stability of EPEC on the infected intestinal mucosa [11, 17, 19]. The EAF plasmid also consists of the plasmid-encoded regulator (per) locus [20] and it has 99% sequence similar to BFP; they have both been reported to cause increased disease outcome in human volunteers [10, 15].

Atypical EPEC lacks the EAF plasmid and, therefore, cannot produce BFP [21]. Atypical EPEC strains are distinguished by their different adherence patterns on cultured epithelial cells. They are becoming increasingly recognized in clinical settings, both in symptomatic and asymptomatic individuals [2, 10], and have also been reported to cause prolonged diarrhea in children [22, 23]. Atypical strains are highly diverse and may cause damage to the host through a variety of ways [10]; therefore, studying the effects of the lack of *bfp* in EPEC infection is important in understanding the disease outcomes. We have recently described an EPEC infection model using weaned mice and showed that EPEC prototype strain E2348/69 was able to colonize the intestine and caused weight decrements, overt diarrhea, intestinal disruption, inflammatory responses, and systemic metabolic perturbations [24]. In this follow-up report, we studied the effects of the prototype EPEC strain (E2348/69) that carries a mutated *bfpA* (site-directed mutant strain UMD901) in the same model to determine whether a non-functional BFP in EPEC leads to altered disease outcomes and compare the results to those of typical EPEC (functional BFP) previously reported [24].

## Methods

### Animal husbandry

Male, 22-day-old C57BL/6 mice utilized in this study were obtained from Jackson Laboratories (Bar Harbor, USA). Upon arrival, mice weighed about 10 g and were co-housed in groups of up to 4 animals per cage. The temperature of the vivarium was regulated between 20 and 23 °C, operating at a 10-h dark and 14-h light cycle. Upon their arrival, mice were given 3 days to acclimate and were placed on standard rodent house chow diet (Harlan), prior and after the infection. Four days before the infection challenge, mice were given an antibiotic cocktail added to the drinking water for 3 days. Mice were then given clean drinking regular water without antibiotics for 24 h as previously described [25]. Throughout the infection period, no antibiotic cocktail was administered. All procedures were equally followed for both infected and uninfected animals.

### EPEC mouse challenge

UMD901 [26] was cultivated in 20 mL Dulbecco’s modified Eagle’s medium (DMEM) containing phenol red and incubated at 37 °C in a shaking incubator until optimal growth of OD_600_ ~ 0.6 was achieved. Cultures were centrifuged at 3500 × *g* for 10 min at 4 °C, and the pellet was resuspended in DMEM high glucose to obtain 10^10^ CFU/mL. Using 22-gauge feeding needles, mice were inoculated with 100 μL of this bacterial suspension by oral gavage. Uninfected mice were gavaged with 100 μL of DMEM high glucose as control.

Throughout the 3-day infection period, all mice were weighed and stools collected daily. After this period, euthanasia of the animals was performed. Intestinal tissues (duodenum, jejunum, ileum, and colon) and cecal contents were collected, flash frozen in liquid nitrogen and kept at − 80 °C until further investigation.

### Stool shedding and tissue burden

DNA was extracted from fresh stool specimens collected at days 1 and 3 post-infection (p.i.) using the QIAamp DNA stool mini kit (Qiagen). DNA from intestinal tissues was extracted using the DNeasy Kit (Qiagen). The *eae* gene with the primer sequences: 5’-CCCGAATTCGGCACAAGCATAAGC-3’ (sense) and 5’-CCCGGATCCGTCTCGCCAGTATTCG-3’ (antisense) [27] was used as the target. Stool shedding and tissue burden were quantified using real-time PCR with the following conditions: 95 °C for 3 min, then 40 cycles for 15 s at 95 °C, 60 s at 55 °C, and 72 °C for 20 s.

### Intestinal inflammatory response

Protein lysates from the stools and cecal contents were extracted using radioimmuno-precipitation assay buffer [25]. After centrifuging the lysates at 8000 × *g* for 5 min, the supernatant was utilized to perform the protein assay using the bicinchoninic acid assay (Thermo Fisher Scientific, USA). Inflammatory biomarkers lipocalin-2 (LCN-2) and myeloperoxidase (MPO) were measured using a commercial ELISA kits (R&D Systems, USA).

### Statistical analysis

Data presented in the study was analyzed using GraphPad prism 8 (GraphPad Inc., USA). All statistical analyses were performed on raw data using two-way ANOVA and Turkey’s post hoc and the Kruskal–Wallis and Dunn’s multiple comparison tests where applicable. Data presented are the mean and standard error of the mean (SEM); differences were considered significant with *p*-value of < 0.05.

## Results

### Non-functional BFP leads to weight loss, bloody stools, and colonization

**Fig. 1:**
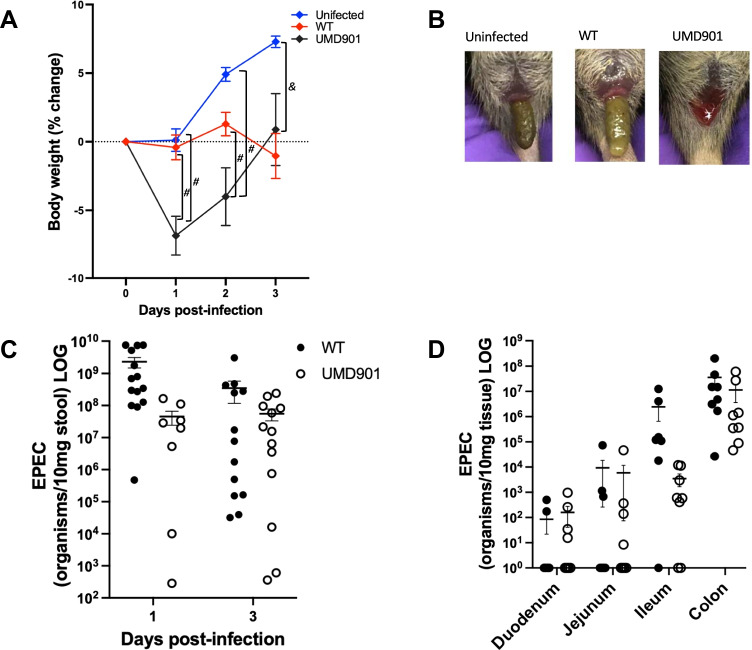
UMD901 (non-functional BFP) affects weight gain, bloody stools accompanied with increased shedding and tissue burden in C57BL/6 mice. **A** Weaned mice pre-treated with antibiotic cocktail were infected with WT EPEC and UMD901; monitored daily for changes in weight (*n* = 12/group). *#p* = 0.003 and *&p* = 0.0004, data analyzed using two-way ANOVA and Turkey’s post hoc test. **B** Change in stool appearance of uninfected and infected (WT and/or UMD901 EPEC) mice. **C** Stool shedding of strain UMD901 vs WT EPEC infected mice. **D** Colonization of WT and UMD901 in different intestinal tissue sections at day 3 p.i. (*n* = 8/group) The uninfected and WT EPEC data has already been published [24] and are included here for comparison with the simultaneously studied strain UMD901 infected mice that are first reported herein

C57BL/6 mice pre-treated with antibiotic cocktail 3 days prior to infection were infected with strain UMD901 (E2348/69 *bfpA* C129S) and monitored daily for changes in weight. The same procedure was performed in parallel with wild-type (WT) EPEC E2348/69 strain, the results of which have been already published elsewhere [24]. For comparison purposes, some of the results already published for this strain have been extracted and included to this report. A significant weight loss of mice infected with strain UMD901 was observed at days 1 and 2 p.i. when compared to uninfected (*p* = 0.003) and WT-infected mice (*p* = 0.003) (Fig. 1A). UMD901-infected mice also had a significant weight change at day 3 p.i. when compared to the uninfected mice (*p* = 0.0004). The effect of strain UMD901 infection in C57BL/6 mice was also assessed based on the consistency and appearance of stools (Fig. 1B). All uninfected mice had well-formed stools. In contrast, 8 of 12 mice infected with strain UMD901 developed bloody stools at days 1 and 2 p.i. and at day 3 p.i. had diarrhea characterized by wet bottom and liquid stools. WT-infected mice also had wet stools, but with no blood. During the infection period, stool specimens were collected and quantified for EPEC shedding using quantitative PCR (Fig. 1C). WT shedding was observed with a mean of 2.3 × 10^9^ organisms/10 mg stool while UMD901 shedding had a mean of 4.5 × 10^7^ organisms/10 mg stool at day 1 p.i.; however, no statistically significant difference was observed. In order to evaluate strain UMD901 colonization, intestinal tissues from the duodenum, jejunum, ileum, and colon were collected and analyzed for tissue burden using PCR following a 3-day infection period (Fig. 1D). We have previously shown that WT EPEC (E2348/69) colonizes across all tissue sections at day 3 p.i. with the highest tissue burden observed in the ileum and colon [24] (data included in Fig. 1D). In this study, strain UMD901 was found to also colonize all tissue sections, with higher tissue burden being observed in the colon.

### Infection with WT and UMD901 strains leads to increased inflammation

**Fig. 2:**
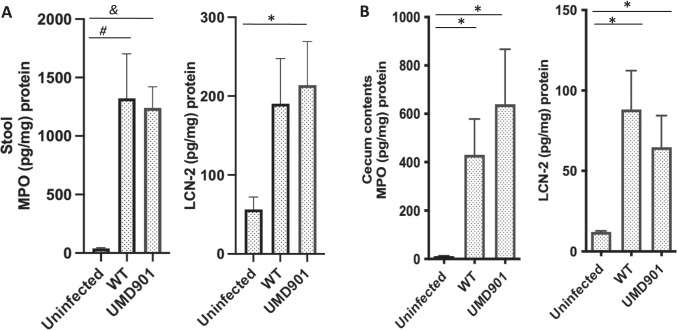
Infection of strain UMD901 in mice causes increased inflammatory biomarkers. **A** Fecal inflammatory biomarkers MPO (*n* = 8 uninfected and WT, 9 UMD901/group) and LCN-2 (*n* = 6 uninfected and UMD901, 5 WT/group) measured in stool specimens collected at day 2 p.i. **B** Inflammatory biomarkers MPO (*n* = 4 uninfected, 7 WT, 8 UMD901/group) and LCN-2 (*n* = 4/group) measured from cecal contents of mice collected at day 3 p.i. Data analyzed using Kruskal–Wallis and Dunn’s multiple comparison test *p = 0.03, *#p* = 0.003, *&p* = 0.0009. As noted in the text, the WT EPEC data has already been published [24], but are included here for comparison with the simultaneously studied strain UMD901-infected mice that are first reported herein

An increase in neutrophil infiltration as a result of intestinal infection is associated with increased biomarkers such as MPO and LCN-2 [28]. Stool specimens collected at day 2 p.i. were analyzed for MPO and LCN-2 inflammatory biomarkers (Fig. 2A). MPO levels of mice infected with WT [24] and UMD901 strains were significantly higher when compared to uninfected mice (*p* = 0.003, *p* = 0.0009) respectively. A significant difference of fecal inflammatory LCN-2 was observed in mice infected with strain UMD901 when compared to the uninfected group (*p* = 0.03) at day 2 p.i. Following euthanasia at day 3 p.i., cecal contents were collected and evaluated for MPO and LCN-2 biomarkers. As seen in Fig. 2B, increased levels of MPO and LCN-2 were also observed to be higher in mice infected with WT and UMD901 strains, when compared to uninfected mice (*p* = 0.03).

## Discussion

The role of EPEC during infection has been reported [14, 29, 30]. Recently, we have developed a murine EPEC infection model using weaned C57BL/6 mice that have been pre-treated with antibiotic cocktail, demonstrating weight alterations, overt diarrhea, intestinal inflammation, and metabolic perturbations [24]. This model also demonstrated that WT EPEC (E2348/69) infection causes watery diarrhea, an important feature that has been reported in humans. Based on the previous EPEC murine model findings, we further evaluated the influence of BFP, which is one of the main traits thought to be involved in typical EPEC localized adherence, microcolony formation, antigenicity, and autoaggregation [10]. Weaned C57BL/6 mice pre-treated with antibiotics were infected with strain UMD901 (a site-directed E2348/69 mutant, presenting a non-functional BFP), and obtained results, compared with the effects of typical WT EPEC previously published [24].

Weaned mice infected with UMD901 exhibited body weight loss, bloody stools, and intestinal inflammation, highlighting the potential relevance of an EPEC strain even without a functioning BFP. The strain UMD901 employed in this study was derived from the same typical EPEC E2348/69 strain and has not gained any additional virulence traits that vary from the WT strain [26]. Mice infected with WT were previously reported to develop moderate to severe diarrhea, with a peak in body weight changes at day 3 p.i., suggesting that higher diarrhea scores are related to greater weight loss [24]. In this study, we observed an early body weight loss at day 1 p.i. accompanied with bloody stool appearance, and the highest colonization was observed in the colon at day 3 p.i. DuPont et al. [29] reported on a *bfpA* (*bfpA*:TnphoA mutant) infection in neonatal mice, and colonization was greater in the small intestines and colon [29], which was comparable to the WT strain [24]. Although DuPont et al. [29] used an EPEC neonate model that does not present clinical symptoms during infection, they demonstrated that BFP required for formation of microcolonies and mucosal innate immune stimulation. In contrast, our data suggest that non-functional BFP had no effect on clinical symptoms or inflammation in our model; this implies that there are relevant differences in antibiotic pre-treated mice, which indicates an interplay between EPEC pathogenesis and microbiota, and this requires further investigation.

*E. coli* pathotypes are constantly evolving and have been shown to acquire/lose virulence traits from one other [31–33]; therefore, the involvement of other virulence strategies in a *bfpA-* background require more investigation. In our study, the strain UMD901 was associated with bloody stools, fecal shedding, and intestinal inflammation that was comparable to that seen with the typical parent EPEC strain in our published studies [24] (performed concurrent with these studies, with strain UMD901). Whether this has relevance to the effects seen with “atypical EPEC” strains in children requires further study with clinical isolates. Bloody diarrhea has not been widely documented in EPEC infections until a recent report of a child developing bloody mucoid diarrhea in India [34]. Other pathotypes of clinical relevance, such as EHEC O157 induce bloody diarrhea associated with HUS [35]. Atypical EPEC strains have additional virulence pathways that might influence disease outcome by rendering them to be symptomatic or asymptomatic in humans [10]. The pathogenicity of atypical EPEC is complex and these strains are frequently found to exhibit virulence genes from various diarrheagenic *E. coli* pathotypes [36, 37]. Atypical EPEC O26 strains have been suggested to be descendants of EHEC O26 that may have lost the Shiga toxin; yet, this EPEC strain has also been linked to bloody diarrhea [33, 38]. Ruiz et al. [37] also reported on an uncommon atypical EPEC strain expressing a plasmid-encoded toxin that has only been documented in EAEC to induce cell damage by altering the cytoskeletal structure. In this model, we therefore predict that the strain UMD901 colonized extensively in multiple intestinal regions, as demonstrated in all the intestinal tissue sections throughout the 3-day infection period, causing increased inflammation and bloody stools. Furthermore, these findings suggest that EPEC infection in mice does not need a functioning *bfpA* for enhanced virulence.

Although comparing animal studies to humans has limitations due to differences in microbiota, colonization of strain UMD901 in all intestinal regions implies that loss of BFP function does not affect broader colonization. These results are in accordance with a previous report by DuPont et al. [29] indicating that *bfpA* is not critical during colonization in neonatal mouse model when compared to WT EPEC. Based on these findings, this could potentially explain the high prevalence of atypical EPEC strains in clinical studies [23, 39, 40]. Bieber et al. [15] previously reported on *bfpA* infection with 2 out of 16 human volunteers developing mild diarrhea and humans infected with the WT EPEC B171-8 strain developing a more severe diarrhea. In conclusion, infection of EPEC without a functional BFP in weaned mice pre-treated with antibiotics causes bloody appearance in stools, accompanied with growth failure and colonization leading to increased inflammatory biomarkers. The potential of further studying atypical EPEC clinical strains in this model is therefore warranted in order to understand the mechanisms of pathogenesis.

## Data Availability

The datasets presented during the current study are available on reasonable request.

## References

[1] Platts-Mills JA, Liu J, Rogawski ET, Kabir F, Lertsethtakarn P, Siguas M, Khan SS, Praharaj I, Murei A, Nshama R (2018). Use of quantitative molecular diagnostic methods to assess the aetiology, burden, and clinical characteristics of diarrhoea in children in low-resource settings: a reanalysis of the MAL-ED cohort study. Lancet Glob Health.

[2] Kotloff KL, Nataro JP, Blackwelder WC, Nasrin D, Farag TH, Panchalingam S, Wu Y, Sow SO, Sur D, Breiman RF (2013). Burden and aetiology of diarrhoeal disease in infants and young children in developing countries (the Global Enteric Multicenter Study, GEMS): a prospective, case-control study. Lancet.

[3] Hartland EL, Leong JM (2013). Enteropathogenic and enterohemorrhagic *E*. *coli*: ecology, pathogenesis, and evolution. Front Cell Infect Microbiol.

[4] Mundy R, Girard F, FitzGerald AJ, Frankel G (2006). Comparison of colonization dynamics and pathology of mice infected with enteropathogenic *Escherichia*
*coli*, enterohaemorrhagic *E*. *coli* and *Citrobacter*
*rodentium*. FEMS Microbiol Lett.

[5] Spears KJ, Roe AJ, Gally DL (2006). A comparison of enteropathogenic and enterohaemorrhagic *Escherichia coli* pathogenesis. FEMS Microbiol Lett.

[6] Schmidt MA (2010). LEEways: tales of EPEC, ATEC and EHEC. Cell Microbiol.

[7] Tennant SM, Tauschek M, Azzopardi K, Bigham A, Bennett-Wood V, Hartland EL, Qi W, Whittam TS, Robins-Browne RM (2009). Characterisation of atypical enteropathogenic *E*. *coli* strains of clinical origin. BMC Microbiol.

[8] Schuller S, Lucas M, Kaper JB, Giron JA, Phillips AD (2009). The ex *vivo* response of human intestinal mucosa to enteropathogenic *Escherichia coli* infection. Cell Microbiol.

[9] Knutton S, Baldini MM, Kaper JB, McNeish AS (1987). Role of plasmid-encoded adherence factors in adhesion of enteropathogenic *Escherichia coli* to HEp-2 cells. Infect Immun.

[10] Gomes TA, Elias WP, Scaletsky IC, Guth BE, Rodrigues JF, Piazza RM, Ferreira LC, Martinez MB (2016). Diarrheagenic *Escherichia coli*. Braz J Microbiol.

[11] Cleary J, Lai LC, Shaw RK, Straatman-Iwanowska A, Donnenberg MS, Frankel G, Knutton S (2004). Enteropathogenic *Escherichia coli* (EPEC) adhesion to intestinal epithelial cells: role of bundle-forming pili (BFP), EspA filaments and intimin. Microbiology (Reading).

[12] Anantha RP, Stone KD, Donnenberg MS (2000). Effects of bfp mutations on biogenesis of functional enteropathogenic *Escherichia coli* type IV pili. J Bacteriol.

[13] Donnenberg MS, Donohue-Rolfe A, Keusch GT (1989). Epithelial cell invasion: an overlooked property of enteropathogenic *Escherichia coli* (EPEC) associated with the EPEC adherence factor. J Infect Dis.

[14] Nataro JP, Maher KO, Mackie P, Kaper JB (1987). Characterization of plasmids encoding the adherence factor of enteropathogenic *Escherichia coli*. Infect Immun.

[15] Bieber D, Ramer SW, Wu CY, Murray WJ, Tobe T, Fernandez R, Schoolnik GK (1998). Type IV pili, transient bacterial aggregates, and virulence of enteropathogenic *Escherichia coli*. Science.

[16] Donnenberg MS, Giron JA, Nataro JP, Kaper JB (1992). A plasmid-encoded type IV fimbrial gene of enteropathogenic *Escherichia coli* associated with localized adherence. Mol Microbiol.

[17] Giron JA, Ho AS, Schoolnik GK (1991). An inducible bundle-forming pilus of enteropathogenic *Escherichia coli*. Science.

[18] Stone KD, Zhang HZ, Carlson LK, Donnenberg MS (1996). A cluster of fourteen genes from enteropathogenic *Escherichia coli* is sufficient for the biogenesis of a type IV pilus. Mol Microbiol.

[19] Knutton S, Shaw RK, Anantha RP, Donnenberg MS, Zorgani AA (1999). The type IV bundle-forming pilus of enteropathogenic *Escherichia coli* undergoes dramatic alterations in structure associated with bacterial adherence, aggregation and dispersal. Mol Microbiol.

[20] Kaper JB (1996). Defining EPEC. Rev Microbiol.

[21] Guerrant RL, Walker DH, Weller PF (2011). Tropical infectious diseases : principles, pathogens and practice.

[22] Nguyen RN, Taylor LS, Tauschek M, Robins-Browne RM (2006). Atypical enteropathogenic *Escherichia coli* infection and prolonged diarrhea in children. Emerg Infect Dis.

[23] Afset JE, Bevanger L, Romundstad P, Bergh K (2004). Association of atypical enteropathogenic *Escherichia coli* (EPEC) with prolonged diarrhoea. J Med Microbiol.

[24] Ledwaba SE, Costa DVS, Bolick DT, Giallourou N, Medeiros P, Swann JR, Traore AN, Potgieter N, Nataro JP, Guerrant RL (2020). Enteropathogenic *Escherichia coli* infection induces diarrhea, intestinal damage, metabolic alterations, and increased intestinal permeability in a murine model. Front Cell Infect Microbiol.

[25] Bolick DT, Medeiros P, Ledwaba SE, Lima AAM, Nataro JP, Barry EM, Guerrant RL (2018) The critical role of zinc in a new murine model of enterotoxigenic *E. coli* (ETEC) diarrhea. Infect Immun 86(7):e00183–e00218 10.1128/IAI.00183-18 PMC6013668 29661930

[26] Zhang HZ, Donnenberg MS (1996). DsbA is required for stability of the type IV pilin of enteropathogenic *Escherichia coli*. Mol Microbiol.

[27] Zhang WL, Kohler B, Oswald E, Beutin L, Karch H, Morabito S, Caprioli A, Suerbaum S, Schmidt H (2002). Genetic diversity of intimin genes of attaching and effacing *Escherichia coli* strains. J Clin Microbiol.

[28] Prata MM, Havt A, Bolick DT, Pinkerton R, Lima A, Guerrant RL (2016). Comparisons between myeloperoxidase, lactoferrin, calprotectin and lipocalin-2, as fecal biomarkers of intestinal inflammation in malnourished children. J Transl Sci.

[29] Dupont A, Sommer F, Zhang K, Repnik U, Basic M, Bleich A, Kuhnel M, Backhed F, Litvak Y, Fulde M (2016). Age-Dependent Susceptibility to Enteropathogenic *Escherichia coli* (EPEC) Infection in Mice. PLoS Pathog.

[30] Savkovic SD, Villanueva J, Turner JR, Matkowskyj KA, Hecht G (2005). Mouse model of enteropathogenic *Escherichia coli* infection. Infect Immun.

[31] Arimizu Y, Kirino Y, Sato MP, Uno K, Sato T, Gotoh Y, Auvray F, Brugere H, Oswald E, Mainil JG (2019). Large-scale genome analysis of bovine commensal Escherichia coli reveals that bovine-adapted *E*. *coli* lineages are serving as evolutionary sources of the emergence of human intestinal pathogenic strains. Genome Res.

[32] Ingle DJ, Tauschek M, Edwards DJ, Hocking DM, Pickard DJ, Azzopardi KI, Amarasena T, Bennett-Wood V, Pearson JS, Tamboura B (2016). Evolution of atypical enteropathogenic *E*. *coli* by repeated acquisition of LEE pathogenicity island variants. Nat Microbiol.

[33] Bielaszewska M, Dobrindt U, Gartner J, Gallitz I, Hacker J, Karch H, Muller D, Schubert S, Alexander Schmidt M, Sorsa LJ (2007). Aspects of genome plasticity in pathogenic *Escherichia coli*. Int J Med Microbiol.

[34] Snehaa K, Singh T, Dar SA, Haque S, Ramachandran VG, Saha R, Shah D, Das S (2021). Typical and atypical enteropathogenic *Escherichia coli* in children with acute diarrhoea: changing trend in East Delhi. Biomed J.

[35] Kaper JB, Nataro JP, Mobley HL (2004). Pathogenic *Escherichia coli*. Nat Rev Microbiol.

[36] Bando SY, Andrade FB, Guth BE, Elias WP, Moreira-Filho CA (2009). Pestana de Castro AF: Atypical enteropathogenic *Escherichia coli* genomic background allows the acquisition of non-EPEC virulence factors. FEMS Microbiol Lett.

[37] Ruiz RC, Melo KC, Rossato SS, Barbosa CM, Correa LM, Elias WP, Piazza RM (2014). Atypical enteropathogenic *Escherichia coli* secretes plasmid encoded toxin. Biomed Res Int.

[38] Hu J, Torres AG (2015). Enteropathogenic *Escherichia coli*: foe or innocent bystander?. Clin Microbiol Infect.

[39] Trabulsi LR, Keller R, Tardelli Gomes TA (2002). Typical and atypical enteropathogenic *Escherichia coli*. Emerg Infect Dis.

[40] Ochoa TJ, Contreras CA (2011). Enteropathogenic *Escherichia coli* infection in children. Curr Opin Infect Dis.

[41] National Research Council (2011) Guide for the care and use of laboratory animals. National Academies Press, Edition 8 1–220

